# Successful Nonoperative Treatment of Isolated Popliteus Tendon Avulsion Fractures in Two Adolescents

**DOI:** 10.1155/2014/759419

**Published:** 2014-08-14

**Authors:** Scott D. McKay, Andrew Holt, Thomas Stout, Viola Qafalijaj Hysa

**Affiliations:** ^1^Texas Children's Hospital, 6701 Fannin Suite 660, Houston, TX 77030, USA; ^2^Baylor College of Medicine, One Baylor Plaza, Houston, TX 77030, USA

## Abstract

Isolated popliteal tendon avulsion fractures are relatively uncommon in the pediatric population as other posterolateral lateral structures are often involved. This report describes two skeletally immature male patients who presented with knee injuries without ligamentous instability and were subsequently diagnosed with isolated popliteus tendon avulsion fractures. Both of these patients were managed nonoperatively and had subjectively full recoveries. As the treatment for isolated popliteal tendon avulsion fractures is still unclear, the report here may contribute to strategies regarding conservative treatment of these injuries.

## 1. Introduction

The posterolateral corner of the knee is a complex structure, consisting of the popliteus tendon, lateral collateral ligament, biceps femoris tendon, popliteal meniscal ligament, popliteal fibular ligament, lateral gastrocnemius muscle, arcuate ligament, the oblique popliteal ligament, and the fabellofibular ligament. Isolated injuries of the popliteus are uncommon, as these injuries are usually associated with damage to other structures including the anterior and posterior cruciate ligaments [1, 2]. According to one study, less than 10% of popliteus injuries are isolated [3]. Isolated popliteus avulsion fractures are particularly uncommon in skeletally immature patients, with only 12 cases reported in children less than 15 years of age [4–11]. It has been postulated that these injuries occur due to the relative weakness of the bone adjacent to the tendon insertion [7]. Due to the infrequency of isolated popliteus tendon avulsion fractures, there is no consensus on treatment method. Some have reported good outcomes nonoperatively [9] while operative management has ranged from removal of the avulsed fragment [12] to reduction and fixation [4, 5, 10]. We present 2 cases successfully managed without surgery.

**Figure 1:**
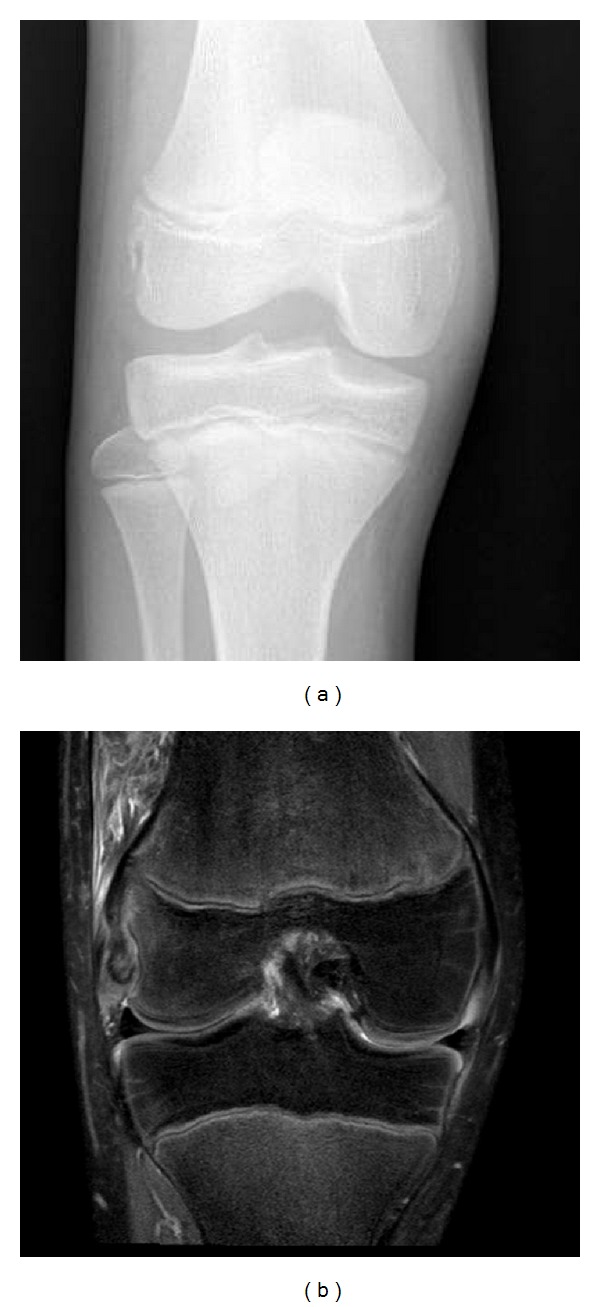
(a) Case 1 injury AP knee showing lateral distal femoral avulsion fracture. (b) Case 1 T2 MRI showing popliteus tendon avulsion.

*Case 1.* A 12-year-old male presented with right knee pain, swelling, and an inability to bear weight. He injured his knee by falling in a dodgeball game, recalling that he heard a “pop” at the time. He had significant right knee effusion with refusal to move the knee secondary to pain on exam. Right knee X-ray showed a nondisplaced avulsion fracture of the right lateral femoral condyle as shown in Figure 1.

Subsequent clinic visits showed moderate effusion, pain with flexion past 90 degrees, and tenderness along the lateral femoral condyle. Examination showed that the right knee had comparable stability to the left, demonstrating only slightly greater opening and translation with varus stress. An MRI confirmed the avulsion fracture of the distal lateral femoral epiphysis at the site of popliteus tendon insertion with partial lateral collateral ligament involvement. Nonoperative treatment was continued, as the patient's knee remained stable on exam. At two weeks post-injury, 15 milliliters of blood was aspirated from the joint space to assist with pain and encourage range of motion.

By seven weeks, the patient was able to run without pain and resume his normal activities. Radiographs of the right knee at that time showed that the avulsion fracture was healing. A follow-up call nearly 2 years after injury reported continued unlimited activities without any complaints.

**Figure 2:**
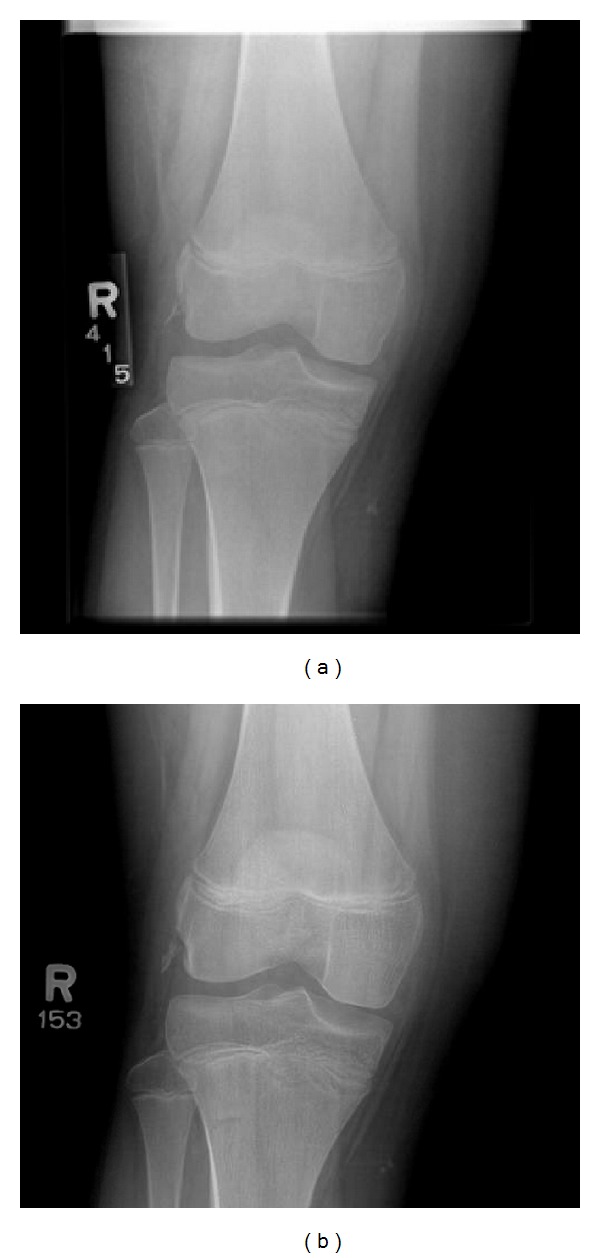
(a) AP radiograph showing avulsion fracture in the area of the popliteus tendon. (b) 6-week follow-up radiograph showing persistent fragment displacement.

*Case 2.* A 12-year-old male was seen in orthopedic clinic with two weeks of right knee pain after a dirt bike injury. He was able to fully bear weight in a splint, which had been placed at an outside hospital. Examination of the right knee showed tenderness of the lateral femoral condyle with no apparent swelling. The knee was stable on exam, comparable to the left side in all aspects. Right knee radiographs and MRI showed a popliteus tendon avulsion fracture. He was treated nonoperatively with a knee immobilizer for comfort and weight bearing as tolerated as shown in Figure 2.

Four weeks after the initial injury, he was able to walk without pain and showed symmetric ligamentous stability on exam. At nine weeks, he was able to run without pain and showed normal gait and knee stability on exam. A follow-up phone call at 6 months after injury revealed the knee had returned completely to preinjury status.

## 2. Discussion

The popliteus muscle-tendon unit is an important structure in the posterolateral compartment of the knee. Its main function is to maintain the dynamic and static posterolateral rotatory stability of the knee joint [13–15]. The popliteus is responsible for “unlocking” the knee joint [4] and for internal rotation of the femur and tibia [5, 16]. It has also been shown to produce an active pivot shift [17]. The anatomy of the muscle is unique in that its distal muscular attachment at the posterior medial aspect of the proximal tibia designated the insertion and the tendinous proximal attachment at the lateral femoral condyle designated the origin. The muscle also has an associated structure called the popliteofibular ligament—a strong tendinous band that attaches to the fibular head and aids in muscle function [18, 19].

The cases presented here were two 12-year-old males with isolated popliteus tendon avulsion fractures. Neither of our patients was able to articulate their exact mechanisms of injury. In previous reports, this injury resulted from knee hyperextension, with the combination of either external rotation of tibia or a varus force on the knee. Both of our patients had symptoms of knee pain with one patient (number 1) having associated swelling, effusion, and guarding. It was unknown whether the other patient (number 2) had these symptoms at the time of injury as he was first seen two weeks after injury. Both patients were treated nonoperatively with a short period of immobilization and weight bearing as tolerated. Aside from an arthrocentesis performed on one patient (number 1) two weeks after injury, no further intervention was required and both patients returned to subjectively normal activity levels within nine weeks. The main weakness of this report is the absence of formal patient outcome assessment to document this complete recovery.

Nonoperative management of isolated popliteal tendon avulsion fractures may be indicated, especially when ligamentous instability is absent. While, in one case series of popliteus tendon avulsion fractures, nonoperative management led to a growth disturbance and valgus deformity that subsequently required surgery, that injury involved the LCL attachment and was a more severe presentation [10]. It is possible that isolated popliteus avulsion fractures may be managed nonoperatively when posterolateral stability is intact, avoiding the risks associated with surgery. Due to the relative infrequency of these injuries, more research on the treatment of isolated posterolateral avulsion fractures is warranted.
